# Ultra-Processed Foods in the Mediterranean Diet according to the NOVA Classification System; A Food Level Analysis of Branded Foods in Greece

**DOI:** 10.3390/foods12071520

**Published:** 2023-04-03

**Authors:** Alexandra Katidi, Antonis Vlassopoulos, Stamoulis Noutsos, Maria Kapsokefalou

**Affiliations:** Laboratory of Chemistry and Food Analysis, Department of Food Science and Human Nutrition, Agricultural University of Athens, 11855 Athens, Greece

**Keywords:** ultra-processed foods, Mediterranean diet, NOVA, branded food composition databases

## Abstract

While the Mediterranean diet (MD) is championed as a healthy and sustainable dietary pattern, the NOVA classification is discussed as a tool to identify ultra-processed foods and further specify healthy food choices. We tested whether the NOVA System aligns with the MD recommendations as presented in the MD pyramids. Foods from the Greek branded food composition database, HelTH, (n = 4581) were scored according to NOVA and assigned to the tiers of the traditional and/or sustainable MD pyramids. Nutritional quality was measured as nutrient content and Nutri-Score grades. NOVA identified 70.2% of all foods as UPFs, and 58.7% or 41.0% of foods included in the sustainable and the traditional MD, respectively. Although foods at the top of the pyramids were mostly (>80%) UPFs, NOVA identified > 50% of foods in the MD base as UPFs. Only 22–39% of foods in the MD base were not UPFs and of high nutritional quality (Nutri-Score A-B). NOVA has low discriminatory capacity across the MD tiers, and it restricts food choices to <30% of foods currently available in supermarkets and <60% within the recommended MD tiers. Therefore, the NOVA classification cannot always identify suitable food choices under the MD pyramid in the modern packaged food environment.

## 1. Introduction

The Mediterranean diet (MD) has been promoted as one of the healthiest dietary patterns because of its association to disease prevention, wellbeing, and longevity [1,2,3]. Moreover, the MD has been championed as a sustainable dietary model as it embodies positive outcomes on environment, food security and nutrition, health, and society [4,5,6,7,8].

The first MD pyramid (MDP) introduced a pictorial summary of the MD guidelines, as they were originally described as part of the Seven Countries Study and the diet of 1960s Crete, Greece [9]. In essence, the MDP assigns food groups in tiers, thus categorizing foods into those that should be consumed daily, weekly, or monthly. Various versions of the MDP describe and synthesize the MD patterns of various countries in the Mediterranean region [10,11,12]. An updated version of the MDP to include sustainability in its graphical illustration has been recently proposed [13]. This MDP recognizes social and technical developments and innovations and consequent changes in the range of foods that are currently available. Therefore, compared to previous MDPs, this MDP embodies the parameter of food processing by presenting its sustainability dimension and by including more processed foods in its pictorial tiers. Nevertheless, it is clearly mentioned that “MD emphasizes the preference for local, seasonal, fresh, and minimally processed foods to enhance their nutritional value and significantly lower the environmental impact of food production, processing, and long-distance import food transportation”.

Food processing is a dimension—in addition to the overall nutritional quality—that should be considered when it comes to the healthiness of foods. The rising consumption of ultra-processed foods (UPFs) has become a public health issue [14,15]. High energy intake from UPFs has been linked to higher risks of obesity, type 2 diabetes, metabolic syndrome, hypertension, cardiovascular disease, depressive symptoms, cancer, and all-cause mortality [16,17,18,19,20,21,22]. It is, therefore, of interest to categorize foods according to the extent and purpose of food processing. For this purpose, the NOVA food classification system has been proposed and used widely [15,23,24,25].

The robustness and functionality of NOVA as a system that helps all stakeholders, including food developers, policymakers, and consumers, to make informed choices in accordance with guidelines, including the MD guidelines, has been questioned but not extensively researched [26]. To work towards filling this knowledge gap, an important element is to understand how the NOVA classification system performs against the MD recommendations, and, specifically, against the categorization of foods in the tiers of the MDP. Understanding how NOVA performs in foods that fall into the MD pattern and are also easily accessed daily by the modern consumer, namely branded foods available to a supermarket, addresses the question in the framework of the current foodscape. This research gap is particularly relevant for the implementation of the MD guidelines in the modern world as a dietary pattern that promotes the consumption of non- and minimally- processed foods at the expense of UPFs. To our best knowledge, no study up to date has addressed how the NOVA system could be applied alongside the MDP in this context.

In this study, we aim to investigate the NOVA classification system in branded foods which could be included in the MD pyramid. To achieve that, (a) we classified branded foods currently sold in Greece according to NOVA, (b) we assessed the NOVA groups distribution in two MD pyramid schemes, and (c) we investigated how differences in NOVA classification were linked to differences in the nutritional composition and profile of similar foods.

## 2. Materials and Methods

### 2.1. Data Source

The Hellenic Food Thesaurus (HelTH), the Greek Branded Food Composition Database (BFCD) [27], was used as the data source for the current study. HelTH is a dynamic repository of food data as presented on-pack, collected through the online sampling of the foods available in the main online supermarkets in Greece, and curated by trained compilers. In its previous version, HelTH contained nutritional composition data for n = 4002 branded foods [27,28,29,30]. HelTH was recently expanded to include 372 pulse products and 477 plant-based meat and dairy imitations [30]. Thus, the current version of the HelTH BFCD used herein consists of 4851 branded food products.

### 2.2. Classification of Foods to the NOVA System Groups

NOVA classifies foods, according to the level and purpose of processing into four distinct groups [23]: (i) NOVA1—unprocessed or minimally processed foods—includes all foods that are directly taken from nature without any processing or with minimal processing or preservation. It includes both animal and plant-based foods that have no added ingredients to them; (ii) NOVA2—culinary ingredients—includes the salt, sugar, oils, and starch that are derived from unprocessed foods or minimally processed foods (e.g., olive oil, flour); (iii) NOVA3—processed foods—includes all foods produced through traditional processing techniques which add culinary ingredients to an unprocessed/minimally processed food (e.g., freshly baked breads, canned vegetables, or cured meats); and (iv) NOVA4—UPFs—includes all ready-to-eat industrially formulated products that include additives and/or substances extracted from foods but contain little to none intact unprocessed/minimally processed ingredients [23,26].

Based on the above definitions, in order for foods to be properly assigned to NOVA categories datasets should include a detailed product description, an ingredient list to identify additives, and an indication of whether a food is industrially formulated. HelTH allows access to the above information, and so ingredient lists were searched for the presence of caloric and/or noncaloric sweeteners in their many forms, added sodium in their many forms, and added oils [23,31,32]. Additional searches were conducted for ≥1 mention of protein isolates or concentrates; for added natural flavors and flavor enhancers; emulsifiers; bulking agents and other thickeners such as sodium carboxymethyl cellulose, cellulose gel, guar gum, xanthan gum, carrageenan, etc. [23,31,32]; and a variety of antioxidants and preservatives and ingredients rarely used in kitchens, such as vitamin A palmitate, vitamin D2, zinc sulfate, sulfur dioxide, etc. [23,31,32].

Products whose ingredient lists could not be acquired were excluded (n = 27). All the remaining products of the HelTH BFCD (n = 4824) were classified into one of the four NOVA Groups. The same methodology was used for the 849 branded foods recently added to the HelTH BFCD.

### 2.3. Classification of Branded Foods under the Mediterranean Diet Pyramid

Two MD pyramid schemes were used for the current analysis.

- The traditional MD Pyramid (tMDP) [9,33] describes the MD as documented in the 1960s in Crete, Greece. The tMDP describes a diet that is rich in unprocessed, fresh, or minimally processed foods. The basis of this diet is formed by fresh, seasonal fruits and vegetables and cereal-based foods that are primarily wholegrain. This diet excludes any novel foods or foods produced with modern processing techniques and it is also based on local produce, i.e., foods produced in Greece.
- The sustainable MD Pyramid (sMDP) [13], which is the most recent revision of the tMDP and has included all traditional Mediterranean foods, along with a variety of modern foods and drinks (such as sodas, sweets, and savory snacks). The sMDP has also included the element of food processing more explicitly within each MDP tier.

Branded foods were screened for inclusion in either or both pyramids and then were assigned to their respective pyramid tier using the same methodology as previously published [34].

### 2.4. Application of the Nutri-Score Algorithm

The Nutri-Score algorithm was calculated for each food based on its nutritional composition per 100 g/mL of food/beverage, as previously described [30,34,35]. Briefly, Nutri-Score studies energy (kJ), total sugars (g), saturated fatty acids (SFAs) (g), and sodium (mg) as “negative nutrients” and scores them in a scale from 0 to 10 for increasing content [36]. On the other hand, protein (g), fiber (g), and fruits/vegetables/pulses/nuts/specific oils (FV%), the “positive nutrients”, are scored from 0 to 5 for increasing content. “Negative” and “positive” nutrient scores are combined to calculate the FSAm-NPS score (Range: −15 to +40) by subtracting the “positive nutrients” score from the “negative nutrients” score [36]. Apart from the numerical FSAm-NPS score, each food is given a Nutri-Score grade from A to E (five-scale Nutri-Score) based on the following criteria [36]: (A) is given to solid foods with FSAm-NPS scores from −5 to −1 and only to waters among beverages, (B) is given to solid foods with FSAm-NPS scores from 0 to 2 and beverages with FSAm-NPS scores from −15 to 1, (C) is given to solid foods with FSAm-NPS scores from 3 to 10 and beverages with FSAm-NPS scores from 2 to 5, (D) is given to solid foods with FSAm-NPS scores from 11 to 18 and beverages with FSAm-NPS scores from 6 to 9 and (E) is given to solid foods with FSAm-NPS scores from 19 to 40 and beverages with FSAm-NPS scores from 10 to 40.

Missing data for any of the “negative nutrients” (energy, saturated fat, total sugar, or sodium) led to an inability to calculate an FSAm-NPS score, and the respective Nutri-Score Grade and such foods were excluded from the analysis (n = 877). On the contrary, missing data for any “positive nutrients” was imputed with zero and FSAm-NPS score and Nutri-Score grade calculations were performed accordingly. Data imputation for “positive nutrients” took place for <10% of foods in food groups where such nutrients are relevant [34]. The main sources of missing nutrient values were lack of nutritional declaration or inability to obtain data due to the low quality of the available images.

### 2.5. Statistical Analysis

Statistical analysis was carried out using IBM SPSS Statistics^®^ (version 23, Northridge, CA, USA). Nutritional composition data (content per 100 g or 100 mL of product) and the FSAm-NPS score were analyzed as continuous variables. Data were tested for normality using the Kolmogorov–Smirnov test. None of the variables followed the normal distribution. Therefore, variables were expressed as median (interquartile range). We assessed the distribution of prepacked products across different NOVA Groups and different Nutri-Score grades for main categories and subcategories overall and per pyramid tiers and per subcategory of traditional foods. Differences were tested using the Kruskal–Wallis non-parametric test for k independent samples. Between-group differences were tested using the Mann–Whitney U test for continuous variables. Statistical significance was set at 0.01% to adjust for multiple comparisons (Bonferroni correction).

## 3. Results

A large proportion of foods currently available in Greek supermarkets do not qualify for inclusion in the MDP. The tMDP was the most restrictive pattern as it only allowed n = 1385 foods (28.3% of HelTH) to be included. Up to n = 2690 foods may be categorized as compatible with the Mediterranean diet under the newly extended sMDP pattern (55.3% of HelTH), which allowed the inclusion of an additional n = 1305 foods as compared to the tMDP. A total of n = 1502 foods (31.0%) were classified as modern foods (e.g., extruded snacks, instant noodles—Asian dishes, soft drinks, energy drinks, etc.) not eligible for inclusion in any version of the MD. A very small portion of foods (2.9%, n = 137) were composite dishes that, despite being traditional and a part of the Mediterranean culinary heritage, could not be clearly mapped in a specific MDP tier and were, therefore, excluded. From the foods most recently added to the HelTH BFCD, pulses could all be mapped in the respective MDP tier in both pyramids, while all plant-based meat and dairy imitations were excluded from both pyramids, as they were not described in any pyramid’s tier specifications.

### 3.1. Distribution of Branded Foods among the NOVA Groups

Table 1 shows the distribution of branded foods available in the Greek market, in the four NOVA Groups for the entire HelTH BFCD and the foods eligible under the tMDP and the sMDP, respectively. From the n = 1385 foods eligible under the tMDP, NOVA classification could be performed for n = 1367. Similarly, from the n = 2690 foods eligible under the sMDP, NOVA classification could be performed for n = 2667 foods. Overall, the MD as a dietary pattern in itself restricts the amount of NOVA4 foods eligible under a healthy MD, excluding 53.7–83.4% of all UPFs currently available in supermarkets. Similar restrictions were seen to a much smaller degree for NOVA1 foods. The tMDP is generally more restrictive in terms of the absolute number of foods eligible than the sMDP. Additionally, foods eligible in the tMDP are more likely to be classified as NOVA1 and less likely to be considered NOVA4 compared to the foods eligible under the sMDP.

**Figure 1:**
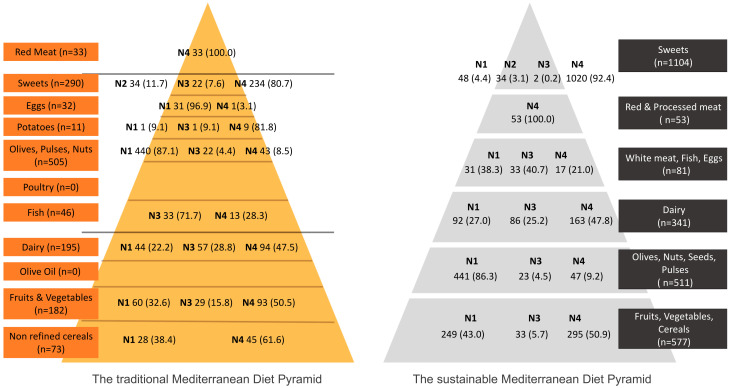
Distribution of the NOVA groups within the traditional and the sustainable Mediterranean diet pyramids’ food tiers. N1 = NOVA1, N2 = NOVA2, N3 = NOVA3, and N4 = NOVA4.

In both pyramids, foods from all four NOVA groups could be found. Almost in every MDP tier, foods from at least two different NOVA groups were seen with the exception of red and processed meats (Figure 1). No other clear pattern of NOVA group clustering could be seen. NOVA 2, which includes culinary ingredients, could only be found in the MDP tier that includes sweets and products that were traditional sweeteners (mainly honey). 

In a branded dataset, all foods considered red or processed meat were classified as NOVA4 or UPFs (Figure 1). The majority of sweets in a supermarket were classified as UPFs in both pyramids. Inside the tiers that compose the bases of the pyramids (non-refined cereals in the tMDP and fruits, vegetables, and cereals in the sMDP) as well as inside the fruits and vegetables tier of the tMDP, over 50% of branded products were classified as UPFs (61.6%, 50.5%, and 50.9%, respectively).

Branded dairy products were most commonly classified as NOVA4 (~48%) under both MDPs. On the contrary, olives, pulses, and nuts (or the equivalent tier) include mainly NOVA1 foods (~86–87%).

Table 2 and Table 3 present the distribution of NOVA groups among the food subcategories that compile the pyramid tiers. It is worth highlighting that the pyramid tiers are usually composed of multiple food subcategories of branded foods with different nutritional compositions (e.g., the dairy tier includes milk, yogurt, and cheese products). In addition, food processing is related to the type of food and, thus, the food subcategory (e.g., cheese is by definition a processed food).

Table 2 showed that for the dairy tier, the NOVA1 group consisted only of milk (63.6%) and yogurt (36.4%) products, the NOVA3 group consisted mainly of cheese products (94.7%), and the NOVA4 group of milk (25.5%), yogurt (37.2%), and cheese (37.2%). For the non-refined cereal tier, the NOVA1 group consisted of rice or similar products (25%) and pasta (75%), while the NOVA4 group consisted mainly of bread or similar products (97.8). Similarly, in the sustainable MDP (Table 3), NOVA1 dairy consisted of milk (73.9%) and yogurt products (26.1%), the NOVA3 group consisted mainly of cheese products (93.0%), and the NOVA4 group of milk (29.5%), yogurt (36.8%), and cheese (33.7%).

### 3.2. Nutritional Quality of Branded Foods Included in the MD Pyramids

Table 4 presents the content of energy (kcal), protein (g), total fat (g), saturated fat (g), total sugars (g), and salt (g) per 100 g/mL of the products, per tier of the tMDP, and per NOVA group. Within the dairy tier, NOVA4 foods had higher energy content than NOVA1 foods, but the highest energy content was seen in NOVA3 foods (*p* < 0.01 for all). However, these differences can be explained by the higher proportion of cheeses in the NOVA3 group compared to the NOVA1 and NOVA4 foods.

For protein, differences were found in the fruits and vegetables (*p* < 0.01), dairy (*p* < 0.01), fish (*p* = 0.002), and olives, pulses, and nuts (*p* < 0.01) tiers. NOVA1 foods were richer in protein than NOVA4 foods in the tiers of olives, pulses, and nuts (*p* < 0.01) and fruits and vegetables (*p* < 0.01). NOVA3 was richer in protein than NOVA4 in the fish (*p* < 0.01), dairy (*p* < 0.01), and fruits and vegetables tiers (*p* < 0.01). In contrast, NOVA1 dairy had a lower content of protein when compared with their NOVA4 counterparts (*p* < 0.01). For total and saturated fat, NOVA1 foods had a lower content than their NOVA4 counterparts in the olives, pulses, and nuts (*p* < 0.01) and non-refined cereals tiers (*p* < 0.01). NOVA1 dairy also had a lower total fat content (*p* = 0.006) than NOVA4. NOVA1 foods had a lower sugar content than their NOVA4 counterparts in the fruits and vegetables tier (*p* = 0.003). NOVA1 foods also had a lower content of salt than their NOVA4 equivalents in the non-refined cereals (*p* < 0.01), fruits and vegetables (*p* < 0.01), dairy (*p* < 0.01), and olives, pulses, and nuts tiers (*p* < 0.01).

A nested analysis inside the pyramid tiers showed that, inside the milk subcategory, differences among NOVA1, NOVA3, and NOVA4 milk products could be found in energy (*p* < 0.01) and total sugars (TS) (*p* < 0.01) (E_NOVA4_ = 72 (56, 127) > E_NOVA1_ = 46 (46, 48), *p* < 0.01, TS_NOVA4_ = 11.9 (5.4, 30.0) > TS_NOVA1_ = 4.7 (4.6, 4.7), *p* < 0.01). Inside the yogurt subcategory, differences between NOVA1 and NOVA4 yogurt products could be found in their sugar content (TS_NOVA4_ = 5.8 (4.5, 11.5) > TS_NOVA1_ = 4.3 (3.8, 4.6), *p* = 0.003). Inside the cheese subcategory, differences between NOVA3 and NOVA4 cheese products could be found in their energy content (E_NOVA4_ = 239 (140, 309) < E_NOVA3_ = 308 (244, 363), *p* = 0.006). Regarding the rest of the food subcategories of the pyramid tiers, differences could be found among NOVA1, NOVA3, and NOVA4 products of the vegetable subcategory of the fruits and vegetables pyramid tier (energy (*p* < 0.01), protein (*p* < 0.01), TS (*p* = 0.001), salt (*p* < 0.01)), the nuts subcategory of the olives, pulses, and nuts pyramid tier (energy (*p* = 0.001), TF (*p* = 0.001), salt (*p* = 0.001)), and between NOVA3 and NOVA4 seeds or kernel products (salt (*p* < 0.01)).

Table 5 presents the content of energy (kcal), protein (g), total fat (g), saturated fat (g), total sugars (g), and salt (g) per 100 g/mL of the products, per tier of the sMDP, and per NOVA group. Differences in the energy value were found in the tier of fruits, vegetable, and cereals, in which NOVA4 had a higher content than NOVA3 food products (*p* = 0.001), and in the tier of dairy, NOVA4 were higher than NOVA1 (*p* < 0.01) but lower in energy than their NOVA3 counterparts (*p* < 0.01). For protein, differences were found in all the sMDP tiers that included more than one NOVA group. Particularly, NOVA4 foods were higher than NOVA1 foods in the dairy (*p* < 0.01) and sweets tiers (*p* < 0.01), but lower in the fruits, vegetables, cereals (*p* < 0.01), and olives, nuts, seeds, and legumes tiers (*p* < 0.01). In addition, NOVA4 olives, nuts, seeds, and legumes products were poorer in protein than their NOVA3 counterparts (*p* = 0.001), while NOVA4 dairy were richer in protein than their NOVA3 counterparts (*p* < 0.01). For total and saturated fat, NOVA4 foods had a higher content from their NOVA1 counterparts in all the sMDP tiers that included more than one NOVA group (*p* < 0.01). NOVA4 foods also had a higher content in total sugars and salt than their NOVA1 counterparts in all the sMDP tiers that included more than one NOVA group (*p* < 0.01).

A nested analysis inside the pyramid tiers showed that differences in energy or macronutrients were identified in the following food subcategories; pasta or similar product, vegetable, nuts, seeds or kernel, milk, yogurt, cheese, seafood product, and juice or nectar. Precisely, for pasta or similar product, differences among NOVA1, NOVA3, and NOVA4 products were found in total fat (*p* < 0.01), SFA (*p* < 0.01), and salt (*p* < 0.01) content. For vegetables, differences among NOVA1, NOVA3, and NOVA4 products were found in energy (*p* < 0.01), protein (*p* < 0.01), sugar (*p* < 0.01), and salt (*p* < 0.01) content. For nuts, differences among NOVA1, NOVA3, and NOVA4 products were found in energy (*p* = 0.001), total fat (*p* = 0.001), and salt (*p* = 0.001) content. For seeds or kernel, differences between NOVA3 and NOVA4 products were found in salt (*p* < 0.01). For milk, differences among NOVA1, NOVA3, and NOVA4 products were found at energy (*p* < 0.01), sugar (*p* < 0.01), and salt (*p* = 0.004) content. For yogurts, differences between NOVA1 and NOVA4 products were found in total fat (*p* < 0.01), SFA (*p* = 0.005), and sugar (*p* < 0.01) content. For cheese, differences between NOVA3 and NOVA4 products were found in salt (*p* = 0.005). For seafood products, differences between NOVA3 and NOVA4 products were found in their protein (*p* = 0.002) and sugar content (*p* < 0.01). For juice or nectar, differences among NOVA1, NOVA3, and NOVA4 products were found in protein (*p* < 0.01).

### 3.3. The NOVA Classification System and Nutri-Score

Table 6 presents the distribution of foods across Nutri-Score grades and their FSAm-NPS score per tMDP tier and NOVA group. Overall, NOVA4 foods had higher FSA-NPS scores than their NOVA1 counterparts (*p* < 0.001). This was only found in the non-refined cereals (*p* < 0.01), fruits and vegetables (*p* < 0.01), dairy (*p* < 0.01), and olives, pulses, and nuts tiers (*p* < 0.01). In the non-refined cereals, fruits and vegetables, and dairy tiers, which are composed of foods with a daily consumption recommendation, NOVA4 foods were graded from A to E, while NOVA1 foods were graded from A to B.

A nested analysis inside the pyramid tiers showed that the FSAm-NPS score differed in the vegetable food subcategory of the fruits and vegetables pyramid tier and the milk and the cheese subcategories of the dairy tier. Precisely, differences were identified among NOVA1, NOVA2, and NOVA4 products of the vegetable subcategory (*p* < 0.01), among NOVA1, NOVA2, and NOVA4 products of the milk subcategory (*p* = 0.009), and between NOVA3 and NOVA4 products of the cheese subcategory (*p* = 0.009).

Table 7 presents the distribution of foods across Nutri-Score grades and their FSAm-NPS score per sMDP tier and NOVA group. NOVA4 foods had a higher FSAm-NPS score than their NOVA1 counterparts (*p* < 0.01). Except for the olives, nuts, seeds, and legumes tier, no NOVA4 food was graded as A or B by the Nutri-Score algorithm; in all the other tiers, NOVA4 foods were graded from A to E. In particular, 80.3%, 78.8%, and 75.5% of the NOVA4 foods from the fruits, vegetables, cereals, dairy and white meat, fish, and eggs tiers, respectively, were graded A–C by Nutri-Score.

A nested analysis inside the pyramid tiers showed that the FSAm-NPS score differed among NOVA groups in the rice or similar grain subcategory (*p* < 0.01), the pasta or similar product subcategory (*p* < 0.01), and the vegetable product subcategory (*p* < 0.01) of the fruits and vegetables tier. For the dairy tier, differences among the FSAm-NPS score of NOVA1, NOVA3, and NOVA4 products were identified for milk (*p* = 0.003) and cheese (*p* = 0.003).

## 4. Discussion

To our best knowledge, this study is the first to directly investigate the agreement of the NOVA classification system with the MD recommendations and, specifically, with the categorization of branded foods available in the Greek market in the tiers of the MDP.

Only 28.3% of the branded foods analyzed fitted in the tMDP, and only 55.3% fitted in the updated sMDP. Traditional Greek foods and dishes including cheese and spinach pies, moussaka, and pasticcio were excluded from both pyramids because they did not directly fit into a specific tier of either pyramid [9,13,34]. Overall, both MDPs showed a tendency to exclude UPFs and promote NOVA1 (minimally processed) foods. Even in the sMDP, which allows for a higher amount of UPFs to be included, UPFs are most likely assigned to the top tier and hence recommended to be consumed sparingly. However, 41% (n = 565) of the branded food products included in the tMDP and 58.7% (n = 1574) of the branded food products included in the sMDP were classified as NOVA4.

In general, branded foods from multiple NOVA groups were seen in almost every MDP tier. Although one would expect NOVA4 (UPFs) to be more common in the top tiers, in fact, both the top and the bottom tiers had the highest concentration of NOVA4 foods. In the tMDP, foods that are typically recommended for daily consumption (bottom tiers) were mainly populated by UPFs when branded foods were considered. Therefore, we could observe that the NOVA classification system could not clearly separate branded foods at the top and the bottom of the pyramids, which was considered an element of poor alignment.

Another interesting finding is that the distribution of NOVA groups inside the pyramid tiers is linked with the food subcategories that compose each tier. Pyramid tiers are created as a visual representation of food groups that should all be consumed with the same frequency (daily, weekly, monthly) and not the homogeneity of the foods in terms of the underlying food composition and chemistry. Therefore, pyramid tiers are usually composed of multiple food subcategories. This means that food subcategories inside a pyramid tier may have differences in their nutritional composition and the manufacturing and/or processing methods employed. Thus, foods included in the same pyramid tier may be distributed in different NOVA groups according to their food subcategory.

Nonetheless, there was a strong relationship between NOVA classification and nutritional composition even within the same MDP tier. In the tMDP, NOVA4 foods were higher in energy, total and saturated fat, total sugars, and salt than their NOVA1 counterparts in the same tier. In the sMDP, differences in energy and/or macronutrients could be found in all pyramid tiers that were composed by more than one NOVA group. NOVA4 foods were always higher in total fat, SFA, total sugars, and salt than their NOVA1 counterparts.

When the nutritional quality of foods was expressed via the Nutri-Score Nutrient Profiling System, there was limited alignment between NOVA and nutritional quality. Even though NOVA1 foods had a lower FSAm-NPS score and, thus, a better nutritional quality than NOVA4, there was a significant percentage of NOVA4 foods graded from A to C. As shown in previous analyses, Nutri-Score tended to increase across the MDP, especially the sMDP, with foods at the bottom being graded mainly as A and D or E at the top [34]. That was irrespective of their degree of processing as shown in the current analysis. These results come in line with recent studies in France, where large percentages of foods highly rated “A” or “B” by Nutri-Score fell into the NOVA4 group [17,31]. This fact poses questions about whether the NOVA classification system provides consumers with contradictory information. Nonetheless, it is clear that NOVA1 foods received better Nutri-Score grading. In fact, only NOVA4 foods were graded as D or E using Nutri-Score without excluding the possibility of identifying NOVA4 foods (UPFs) with Nutri-Score grade A or B.

In contrast, previous studies have proposed that when considering the health-related impact of foods, nutrient profile (e.g., Nutri-Score) and (ultra-)processing are two dimensions that should be considered complementary and not contradictory [37,38,39]. According to a study that compared the nutritional quality of foods, as assessed by Nutri-Score, and the ultra-processing, as assessed by the NOVA classification, UPFs were found in all Nutri-Score categories, ranging from 26.08% in nutritional category A, 51.48% in category B, 59.09% in category C, and 67.39% in category D to up to 83.69% in nutritional category E. This study proposed that the Nutri-Score should be accompanied by complementary labelling indicating the level of processing, such as the NOVA classification [40]. In addition, a randomized controlled trial that included 21,159 participants used a front-of-pack label that combined Nutri-Score with an additional graphic mention that indicated when the food is ultra-processed was carried out. This study demonstrated that participants were able to independently identify and understand these two complementary dimensions of foods compared to a no-label situation [39].

These findings raise concerns about how NOVA could be implemented alongside the MD to promote dietary choices of higher nutritional value. It is a fact that UPF consumption has been inversely associated with adherence to the MD [41,42,43] and overall diet quality [42,44,45,46,47]. Low adherence to the MD has been associated with a higher contribution of UPFs in the diet [41]. In addition, higher adherence to the traditional MD has been associated with lower consumption of UPFs among Spanish children [43]. However, adding NOVA scoring to the MDP does not seem to successfully add a dimension that promotes food choices with better nutritional compositions. In fact, in a foodscape where food processing is abundant, the combination of NOVA and MDP seems to restrict dietary choices drastically for modern consumers. If Nutri-Score is also added to the equation, then food choices with limited processing, good Nutri-Score grading, and at the bottom of the pyramid are extremely scarce in a modern supermarket.

At this point, it is important to draw attention to some of the current study’s limitations. The main drawbacks of this analysis stem either from the nature of the HelTH BFCD and BFCDs in general, or from the nature of the MD pyramid recommendations. As a BFCD, HelTH only includes products that are sold as packaged foods and those that are required by the legislation to include nutritional declaration. When compared to analyses of generic food composition data, using data on branded foods improves the relevance of the results for the consumer and food industry [34]. However, when it comes to the MDP, it also poses unique challenges.

The MD is designed to encourage the eating of locally grown, seasonally available produce that is frequently sold as fresh and unpackaged. For food categories such fruits, vegetables, meat, and fish, a BFCD like HelTH would not be able to map those products, resulting in an underrepresentation of the foods now offered in the market. It is reasonable to assume that most packaged foods in these categories will have undergone more processing than their fresh counterparts, which may have led to an overestimation of the NOVA4 group for each pyramid tier [34].

Despite its limitations, this research is crucial for launching the conversation about whether the NOVA classification system is a useful tool to promote regional dietary recommendations. It is crucial to make sure that the NOVA classification system does not conflict with the MD, which serves as a pillar for both health and agriculture. Diet quality seems more likely to be determined by specific consumer choices from among NOVA4 foods (e.g., NOVA4 foods at the bottom of the MDP and/or graded as A or B by the Nutri-Score) than by a food’s classification to NOVA4 in and of itself [31].

## 5. Conclusions

This study is the first to develop a systematic framework in order to investigate how the NOVA classification system performs against the MD recommendations under a branded food-level analysis. It shows that in the larger scheme, the NOVA classification system shows only partial alignment with the MD dietary recommendations. Therefore, the NOVA classification system cannot always identify suitable food choices under the MD pyramid in the modern packaged food environment. Further research should be conducted to investigate the suitability of the NOVA classification system as a complementary tool to guide food and nutrition policies.

## Figures and Tables

**Table 1:** Distribution of foods included in the traditional Mediterranean diet pyramid (tMDP), in the sustainable Mediterranean diet pyramid (sMDP), and, overall, the HelTH BFCD among the four groups of the NOVA classification system.

	NOVA1 n (%)	NOVA2 n (%)	NOVA3 n (%)	NOVA4 n (%)
HelTH (n = 4824)	983 (20.4)	69 (1.4)	367 (7.6)	3405 (70.6)
tMDP (n = 1367)	604 (44.2)	34 (2.5)	164 (12.0)	565 (41.3)
sMDP (n = 2667)	861 (32.1)	34 (1.3)	177 (7.4)	1595 (58.7)

Values indicate the number and the percentage of products.

**Table 2:** Distribution of NOVA groups among food subcategories of the traditional MD pyramid tiers.

Food Tiers of the Traditional Mediterranean Diet Pyramid	Food Subcategory	NOVA Classification System			
		NOVA1	NOVA2	NOVA3	NOVA4
Red Meat (n = 33)	Preserved Meat (n = 11)	-	-	-	11 (100.0)
	Sausage or similar meat product (n = 21)	-	-	-	21 (100.0)
	Meat dish (n = 1)	-	-	-	1 (100.0)
Sweets (n = 290)	Fine Bakery Ware (n = 92)	-	-	-	92 (100.0)
	Sugar, Honey or Syrup (n = 35)	-	34 (97.1)	1 (2.9)	-
	Jam or Marmalade (n = 69)	-	-	-	69 (100.0)
	Non-chocolate confectionary or other sugar product (n = 64)	-	-	21 (32.8)	43 (67.2)
	Prepared Food Product (n = 30)	-	-	-	30 (100.0)
Eggs (n = 32)	Fresh or Processed Egg (n = 32)	31 (96.9)	-	-	1 (3.1)
Potatoes (n = 11)	Starchy Root or Potato (n = 11)	1 (9.1)	-	1 (9.1)	9 (81.8)
Olives, Pulses, Nuts (n = 505)	Pulses (n = 422)	422 (100.0)	-	-	-
	Nut or Seed Product (n = 12)	5 (41.7)	-	-	7 (58.3)
	Seeds or Kernel (n = 31)	-	-	16 (51.6)	15 (48.4)
	Nuts (n = 38)	12 (31.6)	-	6 (15.8)	20 (52.6)
	Vegetable (n = 1)	1 (100.0)			
	Prepared Food Product (n = 1)	-	-	-	1 (100.0)
Fish (n = 46)	Seafood Product (n = 46)	-	-	33 (71.7)	13 (28.3)
Dairy (n = 195)	Milk (n = 55)	28 (50.9)	-	3 (5.5)	24 (43.6)
	Yogurt (n = 51)	16 (31.4)	-	-	35 (68.6)
	Cheese (n = 89)	-	-	54 (60.7)	35 (39.3)
Fruits and Vegetables (n = 182)	Vegetable (n = 159)	59 (37.1)	-	27 (17.0)	73 (45.9)
	Processed fruit (n = 23)	1 (4.3)	-	2 (8.7)	20 (87.0)
Non refined cereals (n = 73)	Rice or Similar Product (n = 7)	7 (100.0)	-	-	-
	Pasta or Similar product (n = 22)	21 (95.5)	-	-	1 (4.5)
	Bread or similar product (n = 44)	-	-	-	44 (100.0)

**Table 3:** Distribution of NOVA Groups among Food Subcategories of the sustainable MD Pyramid tiers.

Food Tiers of the Sustainable MD Pyramid	Food Subcategory	NOVA Classification System			
		NOVA1	NOVA2	NOVA3	NOVA4
Sweets (n = 1104)	Fine Bakery Ware (n = 278)	-	-	-	278 (100.0)
	Sugar, Honey or Syrup (n = 35)	-	34 (97.1)	1 (2.9)	-
	Jam or Marmalade (n = 69)	-	-	-	69 (100.0)
	Non-chocolate confectionary or other sugar product (n = 64)	-	-	-	64 (100.0)
	Prepared Food Product (n = 40)	-	-	-	40 (100.0)
	Frozen dairy dessert (n = 38)	-	-	-	38 (100.0)
	Cereal or cereal milling product (n = 2)	-	-	-	2 (100.0)
	Chocolate (n = 208)	-	-	-	208 (100.0)
	Juice or Nectar (n = 163)	48 (29.4)	-	1 (0.6)	114 (69.9)
	Non-alcoholic beverages (n = 207)	-	-	-	207 (100.0)
Red and processed Meat (n = 53)	Preserved Meat (n = 31)	-	-	-	31 (100.0)
	Sausage or similar meat product (n = 21)	-	-	-	21 (100.0)
	Meat dish (n = 1)				1 (100.0)
White meat, fish, eggs (n = 81)	Fresh or Processed Egg (n = 32)	31 (96.9)	-	-	1 (3.1)
	Seafood Product (n = 46)	-	-	33 (71.7)	13 (28.3)
	Prepared Food Product (n = 3)	-	-	-	3 (100.0)
Dairy (n = 341)	Milk (n = 122)	68 (55.7)	-	6 (4.9)	48 (39.3)
	Yogurt (n = 84)	24 (28.6)	-	-	60 (71.4)
	Cheese (n = 135)	-	-	80 (59.3)	55 (40.7)
Olives, Pulses, Nuts (n = 511)	Pulses (n = 422)	422 (100.0)	-	-	-
	Nut or Seed Product (n = 12)	5 (41.7)	-	-	7 (58.3)
	Seeds or Kernel (n = 31)	-	-	16 (51.6)	15 (48.4)
	Nuts (n = 38)	12 (31.6)	-	6 (15.8)	20 (52.6)
	Vegetable (n = 7)	2 (28.6)	-	1 (14.3)	4 (57.1)
	Prepared Food Product (n = 1)	-	-	-	1 (100.0)
Fruits, Vegetables, Cereals (n = 577)	Processed fruit (n = 23)	1 (4.3)		2 (8.7)	20 (87.0)
	Vegetable (n = 153)	58 (37.9)	-	26 (17.0)	69 (45.1)
	Starchy Root or Potato (n = 11)	1 (9.1)	-	1 (9.1)	9 (81.8)
	Rice or Similar Product (n = 62)	50 (80.6)	-	-	12 (19.4)
	Pasta or Similar product (n = 162)	139 (85.8)	-	4 (2.5)	19 (11.7)
	Bread or similar product (n = 166)	-	-	-	166 (100.0)

**Table 4:** Nutritional composition of foods that compose the traditional Mediterranean diet pyramid, per pyramid tier and per NOVA group.

Traditional Mediterranean Diet Pyramid													
Traditional MD Pyramid Tiers	NOVA Classification System	Nutritional Composition per 100 g/mL											
		Energy (kcal)	*p*-Value	Protein (g)	*p*-Value	Total Fat (g)	*p*-Value	SFA (g)	*p*-Value	Total Sugars (g)	*p*-Value	Salt (g)	*p*-Value
Red Meat (n = 26)	NOVA4 (n = 26)	210 (140, 302)	n.a.	13.8 (12.2, 17.0)	n.a.	15.5 (5.0, 25.2)	n.a.	5.5 (2.2, 9.7)	n.a.	1.0 (0.5, 1.2)	n.a.	2.40 (2.00, 2.50)	n.a.
Sweets (n = 192)	NOVA3 (n = 10)	359 (312, 526)	0.914	5.9 (0.8, 12.2)	0.692	6.5 (0.1, 33.3)	0.855	0.1 (0.1, 3.7)	0.134	65.0 (35.0, 72.0)	0.01	0.10 (0.00, 0.15)	0.084
	NOVA4 (n = 182)	458 (267, 509)		5.4 (0.9, 8.0)		19.0 (0.3, 26.3)		6.6 (0.0, 10.9)		33.0 (20.1, 49.8)		0.17 (0.03, 0.54)	
Eggs (n = 28)	NOVA1 (n = 27)	138 (138, 150)	n.a.	13.0 (12.6, 13.0)	n.a.	11.0 (10.6, 11.0)	n.a.	3.2 (3.2, 3.2)	n.a.	0.0 (0.0, 0.0)	n.a.	0.29 (0.13, 0.31)	n.a.
	NOVA4 (n = 1)	46 (n.a.)		11.0 (n.a.)		0.3 (n.a.)		0.0 (n.a.)		0.3 (n.a.)		0.44 (n.a.)	
Potatoes (n = 11)	NOVA1 (n = 1)	78 (n.a.)	n.a.	2.4 (2.4, 2.4)	n.a.	0.6 (0.6, 0.6)	n.a.	0.0 (0.0, 0.0)	n.a.	0.8 (0.8, 0.8)	n.a.	0.06 (0.06, 0.06)	n.a.
	NOVA3 (n = 1)	156 (n.a.)		2.5 (2.5, 2.5)		5.9 (5.9, 5.9)		1.0 (1.0, 1.0)		0.8 (0.8, 0.8)		0.06 (0.06, 0.06)	
	NOVA4 (n = 9)	136 (82, 180)		2.2 (1.8, 2.6)		4.0 (1.0, 5.8)		0.7 (0.3, 1.0)		0.9 (0.5, 4.5)		0.25 (0.11, 0.59)	
Olives, Pulses, Nuts (n = 503)	NOVA1 (n = 441)	331 (302, 347)	0.044	22.0 (20.3, 24.6) *	<0.01	1.7 (1.2, 2.4) *	<0.01	0.3 (0.2, 0.5) *	<0.01	2.4 (1.5, 3.7)	<0.01	0.02 (0.00, 0.03) *	<0.01
	NOVA3 (n = 21)	278 (229, 496)		1.6 (1.2, 8.6) *		26.6 (19.4, 36.5)		2.9 (2.4, 4.9)		0.2 (0.0, 0.8)		2.70 (1.62, 3.70)	
	NOVA4 (n = 41)	495 (251, 593)		14.2 (1.9, 20.2)		30.3 (19.9, 49.0)		5.5 (3.0, 7.7)		2.6 (0.1, 5.8)		2.00 (0.10, 4.21)	
Fish (n = 39)	NOVA3 (n = 27)	185 (101, 283)	0.831	20.0 (16.0, 24.0)	0.002	10.7 (0.6, 24.8)	0.975	3.0 (0.2, 4.1)	0.235	0.0 (0.0, 0.1)	<0.01	1.00 (0.95, 1.30)	0.543
	NOVA4 (n = 12)	195 (94, 237)		12.2 (8.5, 14.8)		8.8 (4.4, 14.3)		1.2 (0.5, 2.0)		1.0 (0.8, 1.5)		1,05 (1.00, 1.83)	
Dairy (n = 152)	NOVA1 (n = 36)	47 (46, 70) *	<0.01	3.5 (3.3, 4.4) *	<0.01	1.5 (1.5, 4.0) *	<0.01	1.0 (0.9, 2.0)	<0.01	4.7 (4.3, 4.7)	<0.01	0.10 (0.10, 0.12) *	<0.01
	NOVA3 (n = 35)	308 (201, 357) *		23.0 (16.5, 27.0) *		23.0 (12.0, 28.1) *		15.8 (8.2, 20.0) *		0.6 (0.0, 1.2) *		1.75 (1.30, 2.00) *	
	NOVA4 (n = 81)	96 (69, 202)		6.9 (3.9, 12.2)		3.9 (1.7, 8.4)		2.2 (1.0, 4.8)		4.9 (3.1, 11.6)		0.15 (0.10, 0.76)	
Fruits and Vegetables (n = 169)	NOVA1 (n = 59)	33 (30, 77)	0.078	2.6 (1.7, 4.2) *	<0.01	0.3 (0.2, 0.5)	0.361	0.1 (0.1, 0.1)	0.993	2.2 (1.1, 4.5) *	<0.01	0.03 (0.00, 0.10) *	<0.01
	NOVA3 (n = 21)	85 (63, 102)		4.0 (2.4, 4.4) *		0.4 (0.2, 1.1)		0.1 (0.0, 0.3)		5.2 (3.3, 13.1)		0.40 (0.08, 0.60)	
	NOVA4 n = 89)	32 (25, 258)		1.5 (1.2, 2.1)		0.4 (0.1, 0.2)		0.1 (0.0, 0.2)		3.6 (2.2, 14.8)		0.13 (0.03, 0.63)	
Non refined cereals (n = 56)	NOVA1 (n = 28)	354 (352, 359)	0.016	12.0 (9.3, 12.0)	0.478	1.5 (1.3, 2.0)	<0.01	0.3 (0.2, 0.4)	<0.01	3.3 (1.0, 3.8)	<0.01	0.03 (0.01, 0.05)	<0.01
	NOVA4 (n = 28)	406 (310, 443)		11.0 (9.4, 12.0)		7.5 (3.6, 15.1)		2.8 (0.8, 5.0)		4.5 (3.4, 5.5)		1.10 (0.9, 1.5)	

The table presents only the tiers and NOVA groups that contain at least one food product. Values are presented as median and quartiles Q2 (Q1, Q3). The confidence level was set at 99%. * indicates statistically significant differences (*p* < 0.01) of NOVA1 or NOVA3 vs. NOVA4. n.a.: comparison not applicable due to small sample size (n < 2) in at least one group.

**Table 5:** Nutritional composition of foods that compose the sustainable Mediterranean diet pyramid, per pyramid tier and per NOVA group.

Sustainable Mediterranean Diet Pyramid													
Sustainable MD Pyramid Tiers	NOVA Classification System	Nutritional Composition per 100 g/mL											
		Energy (kcal)	*p*-Value	Protein (g)	*p*-Value	Total Fat (g)	*p*-Value	SFA (g)	*p*-Value	Total Sugars (g)	*p*-Value	Salt (g)	*p*-Value
Sweets (n = 928)	NOVA 1 (n = 48)	48 (46, 52) *	<0.01	0.5 (0.3, 0.6) *	<0.01	0.0 (0.0, 0.1) *	<0.01	0.0 (0.0, 0.0) *	<0.01	10.8 (9.9, 12.0) *	<0.01	0.00 (0.00, 0.00) *	<0.01
	NOVA 3 (n = 11)	340 (283, 519)		1.0 (0.9, 11.5)		2.0 (0.1, 32.3)		0.1 (0.1, 2.8)		64.5 (20.0, 71.8) *		0.10 (0.00, 0.23)	
	NOVA 4 (n = 869)	383 (49, 513)		4.6 (0.3, 7.2)		15.4 (0.0, 28.2)		6.4 (0.0, 14.0)		20.5 (10.6, 41.6)		0.11 (0.01, 0.39)	
Red and processed meat (n = 43)	NOVA 4 (n = 43)	210 (158, 292)	n.a.	13.3 (11.5, 17.3)	n.a.	14.8 (7.8, 23.4)	n.a.	5.1 (2.9, 8.8)	n.a.	0.5 (0.0, 1.0)	n.a.	2.50 (2.11, 2.60)	n.a.
White meat, fish, eggs (n=69)	NOVA 1 (n = 27)	138 (138, 150)	0.031	13.0 (12.6, 13.0)	<0.01	11.0 (10.6, 11.0)	0.973	3.2 (3.2, 3.2)	0.047	0.0 (0.0, 0.0) *	<0.01	0.29 (0.13, 0.31) *	<0.01
	NOVA 3 (n = 27)	185 (101, 283)		20.1 (16.0, 24.0) *		10.7 (0.6, 24.8)		3.0 (0.2, 4.1)		0.0 (0.0, 0.1) *		1.00 (0.95, 1.30)	
	NOVA 4 (n = 15)	195 (90, 306)		12.0 (10.0, 13.0)		9.9 (4.3, 26.0)		1.2 (0.5, 2.1)		1.0 (0.6, 1.6)		1.05 (0.95, 1.77)	
Dairy (n = 289)	NOVA 1 (n = 81)	48 (46, 68) *	<0.01	3.4 (3.3, 3.7) *	<0.01	1.5 (1.5, 3.9) *	<0.01	1.1 (0.9, 2.3) *	<0.01	4.7 (4.5, 4.7) *	<0.01	0.10 (0.10, 0.12) *	<0.01
	NOVA 3 (n = 63)	282 (186, 364) *		23.5 (16.4, 27.0) *		23.0 (13.0, 28.4) *		15.5 (9.2, 20.0) *		0.5 (0.0, 0.9) *		1.75 (1.3, 2.2) *	
	NOVA 4 (n = 145)	96.0 (65.5, 168.5)		6.7 (3.8, 11.0)		3.8 (1.6, 8.9)		2.2 (1.0, 5.4)		5.2 (3.8, 10.6)		0.15 (0.10, 0.78)	
Olives, nuts, seeds, legumes (n = 476)	NOVA 1 (n = 412)	331 (302, 347)	0.049	22.0 (20.3, 24.6) *	<0.01	1.7 (1.2, 2.4) *	<0.01	0.3 (0.2, 2.4) *	<0.01	2.4 (1.5, 3.7)	<0.01	0.02 (0.00, 0.03) *	<0.01
	NOVA 3 (n = 22)	278 (211, 492)		1.6 (1.2, 6.5) *		25.9 (17.9, 36.3)		2.9 (2.4, 4.8)		0.4 (0.4, 1.0)		2.70 (1.54, 3.64)	
	NOVA 4 (n = 42)	493 (245, 592)		13.6 (1.9, 19.9)		30.1 (15.5, 49.0)		5.5 (3.0, 7.8)		2.6 (0.1, 5.8)		2.00 (0.10, 4.21)	
Fruits, Vegetables, Cereals (n = 497)	NOVA 1 (n = 238)	354 (152, 358)	0.005	11.5 (5.2, 12.0) *	<0.01	1.5 (0.6, 2.0) *	<0.01	0.3 (0.2, 0.4) *	<0.01	3.0 (1.7, 3.5) *	<0.01	0.02 (0.01, 0.05) *	<0.01
	NOVA 3 (n = 25)	96 (73, 120) *		4.2 (2.7, 5.3)		0.5 (0.2, 1.8) *		0.1 (0.0, 0.5) *		4.9 (3.3, 12.7)		0.45 (0.08, 0.70)	
	NOVA 4 (n = 234)	292 (93, 406)		8.9 (2.0, 11.4)		4.0 (0.5, 10.0)		1.0 (0.1, 3.0)		3.6 (2.0, 5.5)		0.85 (0.20, 1.42)	

The table presents only the tiers and NOVA groups that contain at least one food product. Values are presented as median and quartiles Q2 (Q1, Q3). The confidence level was set at 99%. * indicates statistically significant differences (*p* < 0.01) of NOVA1 or NOVA3 vs. NOVA4. “n.a.: comparison not applicable due to small sample size (n < 2) in at least one group.

**Table 6:** The NOVA classification system, FSAm-NPS score, and distribution of the Nutri-Score grades across the traditional MD pyramid’s tiers.

Traditional Mediterranean Diet Pyramid								
Tiers of the Traditional Mediterranean Diet Pyramid	NOVA Classification System	Nutri-Score						
		Score	*p*-Value	A [n (%)]	B [n (%)]	C [n (%)]	D [n (%)]	E [n (%)]
Red Meat (n = 23)	NOVA 4 (n = 23)	16.174 ± 4.418	n.a.	-	-	1 (4.3)	14 (60.9)	8 (34.8)
Sweets (n = 168)	NOVA 3 (n = 7)	10.714 ± 5.736	0.068	1 (14.3)	-	-	6 (85.7)	-
	NOVA 4 (n = 161)	15.522 ± 6.824		4 (2.5)	3 (1.9)	22 (13.7)	69 (42.9)	63 (39.1)
Eggs (n = 27)	NOVA 1 (n = 26)	−0.500 ± 0.860 *	n.a.	10 (38.5)	16 (61.5)	-	-	-
	NOVA 4 (n = 1)	−4.000		1 (100.0)	-	-	-	-
Potatoes (n = 11)	NOVA 1 (n = 1)	−2.000	n.a.	1 (100.0)	-	-	-	-
	NOVA 3 (n = 1)	−3.000		1 (100.0)	-	-	-	-
	NOVA 4 (n = 9)	−0.222 ± 1.394		3 (33.3)	6 (66.7)	-	-	-
Olives, Pulses, Nuts (n = 312)	NOVA 1 (n = 253)	−1.103 ± 3.872 *	<0.01	195 (77.1)	35 (13.8)	17 (6.7)	4 (1.6)	2 (0.8)
	NOVA 3 (n = 19)	11.947 ± 5.126 *		-	1 (5.3)	6 (31.6)	11 (57.9)	1 (5.3)
	NOVA 4 (n = 40)	12.400 ± 4.187		-	-	14 (35.0)	24 (60.0)	2 (5.0)
Fish (n = 34)	NOVA 3 (n = 23)	6.000 ± 6.00	0.293	1 (4.3)	7 (30.4)	7 (30.4)	8 (34.8)	-
	NOVA 4 (n = 11)	3.818 ± 4.490		1 (9.1)	5 (45.5)	4 (36.4)	1 (9.1)	-
Dairy (n = 128)	NOVA 1 (n = 30)	−0.700 ± 1.119 *	<0.01	20 (66.7)	10 (33.3)	-	-	-
	NOVA 3 (n = 30)	13.267 ± 5.166 *		2 (6.7)	-	2 (3.5)	25 (83.3)	1 (3.3)
	NOVA 4 (n = 68)	4.206 ± 7.049		22 (32.4)	23 (33.8)	8 (11.8)	13 (19.1)	2 (2.9)
Fruits and Vegetables (n = 155)	NOVA 1 (n = 57)	−6.597 ± 2.802 *	<0.01	56 (98.2)	1 (1.8)	-	-	-
	NOVA 3 (n = 21)	−4.619 ± 2.081 *		21 (100.0)	-	-	-	-
	NOVA 4 (n = 77)	1.143 ± 10.782		50 (64.9)	10 (13.0)	5 (6.5)	1 (1.3)	11 (14.3)
Non refined cereals (n = 56)	NOVA 1 (n = 28)	−2.464 ± 2.064 *	<0.01	26 (92.9)	2 (7.1)	-	-	-
	NOVA 4 (n = 28)	7.429 ± 8.153		10 (35.7)	1 (3.6)	7 (25.0)	7 (25.0)	3 (10.7)

The table presents only the tiers and NOVA groups that contain at least one food product. The confidence level was set at 99%. * indicates statistically significant differences (*p* < 0.05) of NOVA1 or NOVA3 vs. NOVA4. n.a.: comparison not applicable due to small sample size (n < 2) in at least one group.

**Table 7:** The NOVA classification system, FSAm-NPS score, and distribution of the Nutri-Score grades across the sustainable MD pyramid’s tiers.

Sustainable Mediterranean Diet Pyramid								
Tiers of the Sustainable Mediterranean Diet Pyramid	NOVA Classification System	Nutri-Score						
		Score	*p*-Value	A [n (%)]	B [n (%)]	C [n (%)]	D [n (%)]	E [n (%)]
Sweets (n = 870)	NOVA 1 (n = 48)	9.958 ± 2.073 *	<0.01	-	-	2 (4.2)	17 (35.4)	29 (60.4)
	NOVA 3 (n = 8)	9.750 ± 5.970 *		1 (12.5)	-	1 (12.5)	6 (75.0)	-
	NOVA 4 (n = 814)	15.491 ± 7.792		5 (0.6)	58 (7.1)	72 (8.8)	223 (27.4)	456 (56.0)
Red and processed meat (n = 39)	NOVA 4 (n = 39)	16.385 ± 3.958	n.a.	-	-	1 (2.6)	24 (61.5)	14 (35.9)
White meat, fish, eggs (n = 62)	NOVA 1 (n = 26)	−0.500 ± 0.860 *	<0.01	10 (38.5)	16 (61.5)	-	-	-
	NOVA 3 (n = 23)	6.000 ± 6.000		1 (4.3)	7 (30.4)	7 (30.4)	8 (34.8)	-
	NOVA 4 (n = 13)	4.385 ± 6.384		2 (15.4)	5 (29.4)	4 (30.8)	1 (7.7)	1 (7.7)
Dairy (n = 251)	NOVA 1 (n = 68)	−0.427 ± 1.189 *	<0.01	39 (57.4)	29 (42.6)	-	-	-
	NOVA 3 (n = 55)	13.655 ± 5.247 *		4 (7.3)	-	4 (7.3)	42 (76.4)	5 (9.1)
	NOVA 4 (n = 128)	4.156 ± 7.047		35 (27.3)	52 (40.6)	14 (10.9)	23 (18.0)	4 (3.1)
Olives, nuts, seeds, legumes (n = 514)	NOVA 1 (n = 254)	−1.126 ± 3.88 *	<0.01	196 (77.2)	35 (13.8)	17 (6.7)	4 (1.6)	2 (0.8)
	NOVA 3 (n = 20)	11.300 ± 5.768		1 (5.0)	1 (5.0)	6 (30.0)	11 (55.0)	1 (5.0)
	NOVA 4 (n = 40)	12.400 ± 4.187		-	-	14 (35.0)	24 (60.0)	2 (5.0)
Fruits, Vegetables, Cereals (n = 464)	NOVA 1 (n = 221)	−3.860 ± 2.744 *	<0.01	214 (96.8)	5 (2.3)	2 (0.9)	-	-
	NOVA 3 (n = 25)	−2.560 ± 5.621 *		21 (84.0)	1 (4.0)	2 (8.0)	1 (4.0)	-
	NOVA 4 (n = 218)	3.826 ± 8.657		89 (40.8)	39 (17.9)	47 (21.6)	26 (11.9)	17 (7.8)

The table presents only the tiers and NOVA groups that contain at least one food product. The confidence level was set at 99%. * indicates statistically significant differences (*p* < 0.05) of NOVA1 or NOVA3 vs. NOVA4. n.a.: comparison not applicable due to small sample size (n < 2) in at least one group.

## Data Availability

HelTH is available at https://www.eurofir.org/our-tools/foodexplorer/ (accessed on 26 January 2023).
